# Computational exploration of palmitoyl-protein thioesterase 1 inhibition by *Juniperus phoenicea* L. for anti-dementia treatment

**DOI:** 10.1016/j.jtumed.2024.12.005

**Published:** 2024-12-12

**Authors:** Riyan A. Putera Irsal, Gusnia Meilin Gholam, Maheswari Alfira Dwicesaria, Tiyara F. Mansyah, Fernanda Chairunisa

**Affiliations:** a Departement of Curriculum and Research, Biomatics, Bogor, West Java, Indonesia; b Department of Biochemistry, Faculty of Mathematics and Natural Sciences, Bogor Agricultural University, Bogor, Indonesia; c Bioinformatics Research Center, Indonesian Institute of Bioinformatics, Malang, Indonesia; d Universitas Nasional, Department of Biology, South Jakarta, Indonesia

**Keywords:** الطب البديل, الإرساء, الديناميكا الجزيئية, التنبؤ الحاسوبي, السمية, Alternative medicine, Docking, Molecular dynamics, PASS server, Toxicity

## Abstract

**Objectives:**

Dementia, a growing concern globally, affects more than 55 million people—a number projected to rise to 152 million by 2050. Current medications target Alzheimer's disease, the most prevalent form of dementia. This study investigated *Juniperus phoenicea* L., a plant used in traditional Chinese medicine, as a potential inhibitor of palmitoyl-protein thioesterase 1 (PPT1), an enzyme associated with dementia.

**Methods:**

*J. phoenicea* phytochemicals were subjected to *in silico* docking against PPT1 (PDB ID: 1EH5). Docking simulations were performed in YASARA Structure with VINA scoring. Top-ranked ligands were subjected to ADMET analysis (admetlab 2.0, Protox 3.0) and PASS bioactivity prediction. Stability and reactivity were analyzed with DFT calculations (Gaussian 09), and 500 ns MD simulations (YASARA Structure, AMBER 14 force field) to assess protein-ligand complex stability. MM-PBSA was used to calculate binding free energies.

**Results:**

The docking simulations identified amentoflavone (−9.6 kcal/mol) as the top hit, followed by ferruginol and quercetin 3-*O*-pentoside. Amentoflavone formed the most interactions (19) with PPT1. *In silico* toxicity analysis predicted amentoflavone and quercetin 3-*O*-pentoside to be safe, whereas ferruginol violated the Pfizer rule. The PASS server indicated a higher probability of activity for quercetin 3-*O*-pentoside (0.423) than amentoflavone (0.287) for dementia treatment. DFT calculations revealed similar electronic properties for both ligands, although amentoflavone showed slightly more favorable values. MD simulations demonstrated that amentoflavone, compared with to galantamine, had superior binding stability in the PPT1 binding pocket.

**Conclusion:**

This *in silico* study was aimed at identifying potential inhibitors of PPT1 from *J. phoenicea* phytochemicals, given that PPT1 is a target for developing new dementia medications. Our findings identified amentoflavone as a promising candidate for further investigation. These findings warrant further research to validate this compound's potential as a PPT1 inhibitor for dementia treatment.

## الملخص

**أهداف البحث:**

يمثل الخرف مشكلة عالمية متزايدة، حيث يؤثر على أكثر من 55 مليون شخص ومن المتوقع أن يرتفع إلى 152 مليون بحلول عام 2050. تستهدف الأدوية الحالية مرض الزهايمر، وهو أكثر أشكال الخرف شيوعا. تبحث هذه الدراسة في نبات العرعر الفينيقي، المستخدم في الطب الصيني التقليدي، كمثبط محتمل لإنزيم ثيوإستراز البروتين البالميتويلي 1، وهو إنزيم مرتبط بالخرف.

**طرق البحث:**

استخدمت هذه الدراسة المركبات النباتية من العرعر الفينيقي للإرساء الحاسوبي ضد الإنزيم المستهدف. تم إجراء محاكاة الإرساء باستخدام برامج متخصصة. خضعت المركبات الأعلى تصنيفا لتحليل الامتصاص والتوزيع والأيض والإفراز والسمية، وتنبؤ النشاط البيولوجي. درست حسابات نظرية الكثافة الوظيفية الاستقرار والتفاعلية. قيمت محاكاة الديناميكا الجزيئية استقرار معقد البروتين-المركب لمدة 500 نانوثانية. تم حساب طاقات الارتباط الحرة.

**النتائج:**

حددت محاكاة الإرساء مركب الأمنتوفلافون كأفضل مركب، يليه الفيروجينول وكيرستين بنتوسيد. شكل الأمنتوفلافون أكبر عدد من التفاعلات مع الإنزيم. توقع تحليل السمية الحاسوبي أن الأمنتوفلافون وكيرستين بنتوسيد آمنان، بينما خالف الفيروجينول القواعد المطلوبة. أشارت التحليلات إلى احتمالية أعلى لنشاط كيرستين بنتوسيد مقارنة بالأمنتوفلافون لعلاج الخرف. كشفت الحسابات النظرية عن خصائص إلكترونية متشابهة لكلا المركبين، مع قيم مواتية قليلا للأمنتوفلافون. أظهرت محاكاة الديناميكا الجزيئية استقرارا أفضل لارتباط الأمنتوفلافون مقارنة بالجالانتامين في موقع ارتباط الإنزيم.

**الاستنتاجات:**

هدفت هذه الدراسة الحاسوبية إلى تحديد مثبطات محتملة للإنزيم من المركبات النباتية للعرعر الفينيقي، حيث يعد الإنزيم هدفا لتطوير أدوية جديدة للخرف. تسلط نتائجنا الضوء على الأمنتوفلافون كمركب واعد يستحق مزيدا من البحث. تستدعي هذه النتائج إجراء المزيد من الدراسات للتحقق من إمكاناته كمثبط للإنزيم لعلاج الخرف.

## Introduction

Globally, dementia currently affects more than 55 million individuals, more than 60 % of whom are from low- and middle-income countries.^1^^,^^2^ This figure is increasing annually, with nearly 10 million new cases reported; the number of affected individuals is projected to rise from 55 million to 82 million by 2030 and to reach 152 million by 2050, given the increasing proportions of older individuals in almost all countries.^3^ Dementia remains a major public health concern worldwide necessitating further treatment development.^4^ Currently, memantine and donepezil, the most frequently prescribed medications for dementia, target the symptoms of Alzheimer's disease (AD), the most prevalent form of dementia.^5^^,^^6^ Rivastigmine and galantamine have also been approved in the UK to help alleviate symptoms in individuals with dementia, including impaired memory and thinking.^7^ Currently, the search for inhibitors of dementia and AD continues in many countries worldwide.^6^ However, use of natural ingredients for drug development for dementia is still limited.

In traditional Chinese medicine (TCM), the genus *Juniperus* is used to prevent or treat various ailments. The plant genus *Juniperus* L, represented by 70 species worldwide and 23 species in China, belongs to the Cupressaceae family.^8^, ^9^, ^10^ Various species within the genus *Juniperus* possess a wealth of phytochemicals such as diterpenes, triterpenes, phenolic acids, flavonoids, and lignans.^11^^,^^12^ The plants’ high phytochemical content directly influences their biological activity; consequently, several *Juniperus* species have been reported to exhibit antimicrobial, antioxidant, antitumor, and anticancer effects.^13^, ^14^, ^15^, ^16^ Specifically, *Juniperus phoenicea* L. acts as a neuroprotective agent inhibiting acetylcholinesterase activity.^17^^,^^18^

Palmitoyl-protein thioesterase 1 (PPT1), a member of the depalmitoylation enzyme group, has been linked to dementia-related conditions, particularly neurodegenerative diseases affecting adults, including AD and frontotemporal dementia.^19^^,^^20^ Notably, this enzyme is critical in neurodevelopmental morphological processes and synaptic function within mature cells. The dysregulation of protein palmitoylation in adults, which is central to neurodegenerative diseases such as AD, has emerged as an important pathogenic mechanism underlying these disorders.^21^ Consequently, genetic mutations affecting the enzymes involved in both palmitoylation and depalmitoylation frequently result in the development of neurological disorders.^22^^,^^23^ These mutations disrupt finely tuned protein modification processes, and consequently contribute to the pathogenesis of various neurological conditions. Understanding the effects of the inhibitory activity of molecules on this enzyme is essential for drug development and enhancing targeted therapeutic strategies to mitigate their effects. Hence, further investigation of the role of *J. phoenicea* as an inhibitor of the PPT1 enzyme is necessary to advance dementia drug development.

## Materials and Methods

### Retrieval, validation, and preparation of proteins and ligands

**Figure 1:**
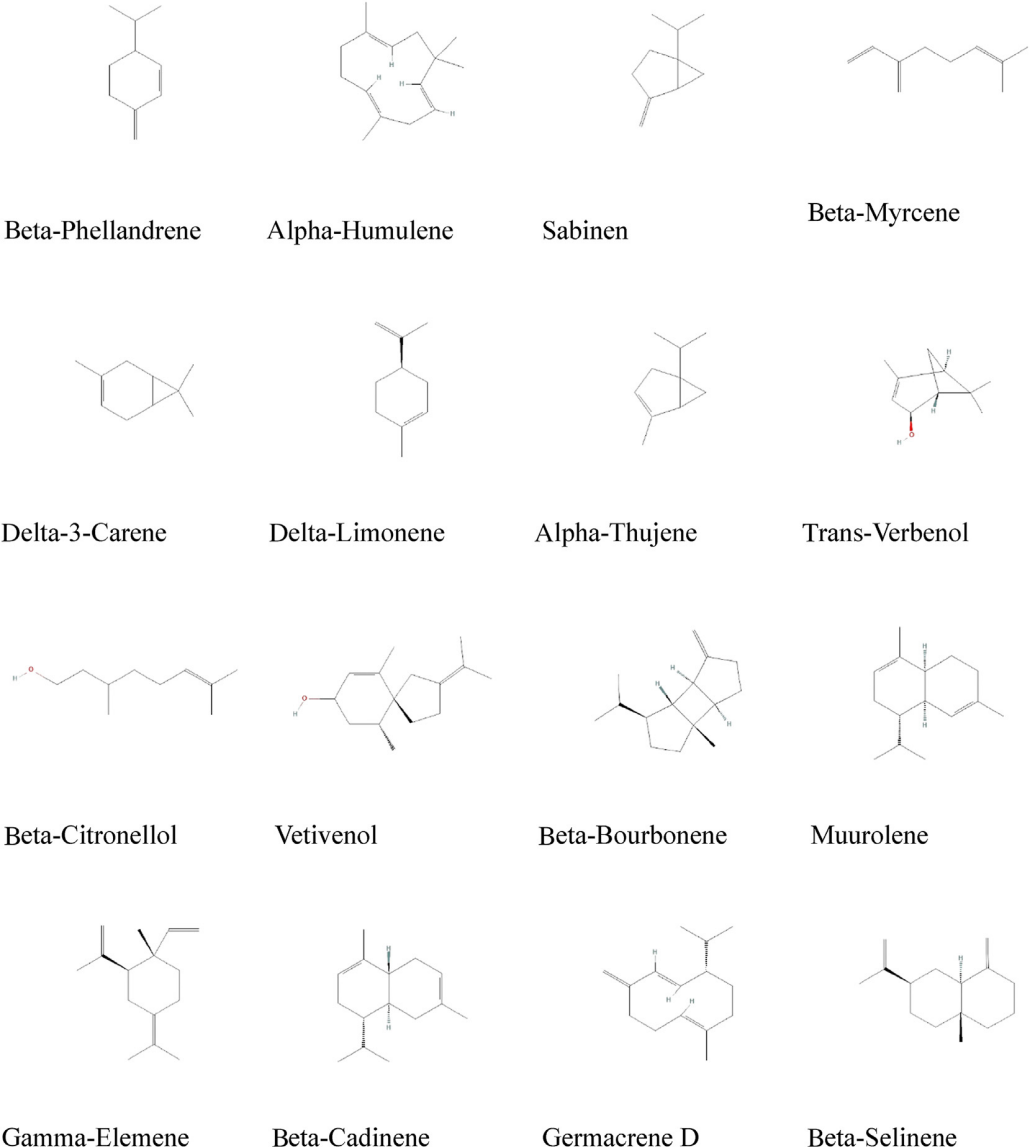

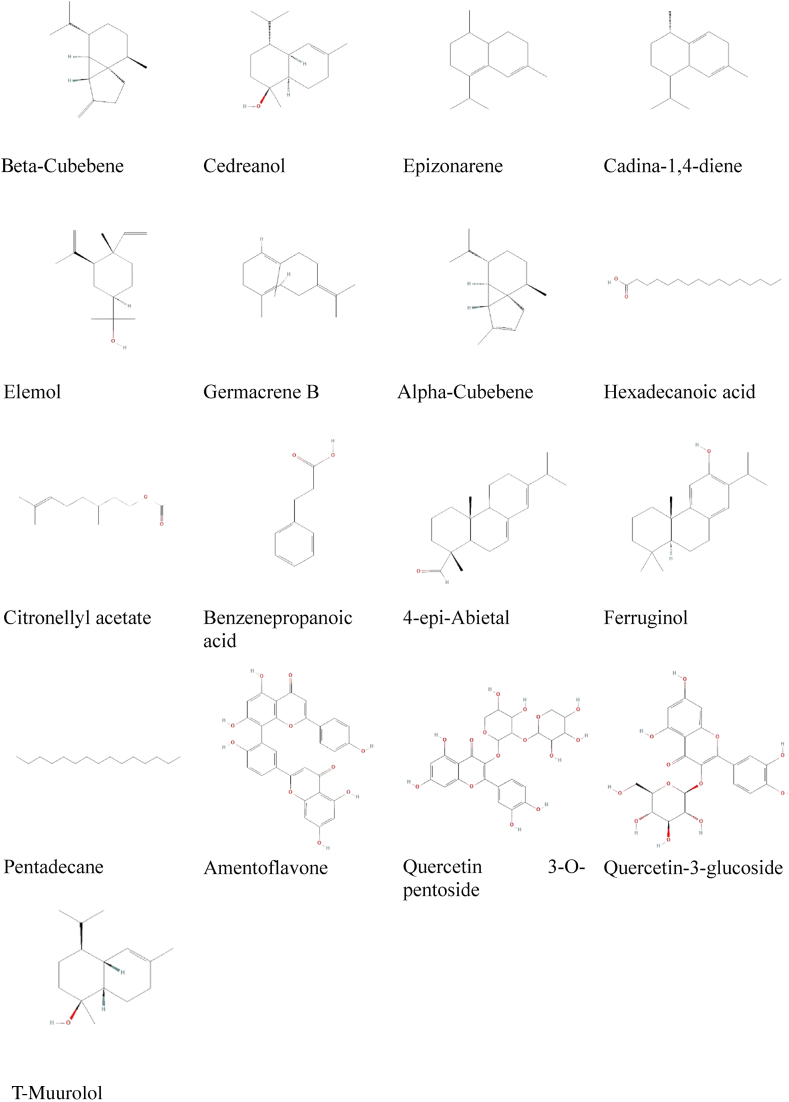
Phytochemicals identified from *Juniperus phoenicea* L.

A crystal structure of the PPT1 receptor (PDB ID: 1EH5; resolution: 2.50 Å) was retrieved from the Protein Data Bank (PDB; https://www.rcsb.org/). The structure was prepared for docking simulations in YASARA Structure (Bioinformatics 30, 2981–2982). Water molecules and nonessential residues were removed, and hydrogen were added. Subsequently, bond orders and hydrogens were adjusted to reflect a physiological pH of 7.4 in YASARA (options > set default pH > 7.4>OK). The CavityPlus server (http://www.pkumdl.cn:8000/cavityplus/#/computation) was used to identify potential ligand-binding pockets within the prepared protein structure. This server uses a structural geometry-based approach for pocket detection and analysis.^24^ The quality of the prepared enzyme structure was evaluated with a suite of structural validation tools available on the SAVES server, including PROCHECK, ERRAT, PROVE, and Verify3D (https://saves.mbi.ucla.edu/).^25^ We used *J. phoenicea* as a source of phytochemicals. Identification of these phytochemicals was guided by findings reported by Kekes et al..^26^ The two-dimensional structures of phytochemicals or ligands were retrieved from PubChem (https://pubchem.ncbi.nlm.nih.gov/) (Figure 1). All candidate ligands underwent energy minimization following ligand preparation in YASARA Structure software. This process optimized the atomic positions within each ligand molecule, thus yielding the structures with the lowest potential energy.

### Molecular docking

The prepared phytochemicals were used to perform molecular docking studies against PPT1 protein to identify potential ligands for further drug discovery experiments. Docking was conducted in YASARA Structure with the dock_runscreening macro. We selected the VINA method and set the number of runs to 100 for each ligand. This method can be interpreted as the sum of the intermolecular part of the lowest-scoring conformation and the intramolecular contributions. In the docking process, we applied the method to PPT1 (PDB ID: 1EH5) by using a grid size of X = 22.43 Å, Y = 22.43 Å, and Z = 22.43 Å, or cuboid grid box = 3 Å. After the docking simulations, the results were saved in .pdb format to facilitate visualization and analysis. Discovery Studio 2017 R2 Client was used for post-docking analysis focusing on the structural interactions of the top-ranked ligands within each enzyme. This analysis enabled us to refine the initial set of 33 phytocompounds, two control drugs (memantine and galantamine), and one native ligand (palmitic acid). On the basis of the binding energy rankings, we narrowed our selection to the three most promising phytocompounds and one control drug for further investigation.^27^^,^^28^

### Pfizer rule and toxicity prediction

For further analysis, we analyzed the toxicity of the three most favorable phytochemicals in the molecular docking by using admetlab 2.0 (https://admetmesh.scbdd.com/service/evaluation/index) and Protox 3.0 (https://comptox.charite.de/protox3/). On the basis of lipophilicity (log P) and topological polar surface area (TPSA), we used the Pfizer rule to evaluate potential drug-likeness and minimize off-target effects. Protox 3.0 predicted a wide range of toxicity endpoints, including LD_50_, toxicity class, hepatotoxicity, neurotoxicity, mutagenicity, cytotoxicity, and carcinogenicity. Notably, SMILES strings were used to facilitate toxicity predictions within both platforms. Ideally, drug candidates should exhibit minimal predicted toxicity in humans. Therefore, any phytochemicals violating the Pfizer rule or exceeding predefined toxicity thresholds in Protox 3.0 were excluded from further investigation.^29^^,^^30^

### Prediction of probability of being active

The selected phytochemicals were further evaluated for their potential bioactivity with the PASS server (http://www.way2drug.com/PASSonline/). The canonical SMILES strings of each ligand were submitted to the server, and dementia treatment was selected as the target parameter. The server predicted the probability of each phytochemical being active (Pa) and inactive (Pi).^31^

### Density functional theory *in silico* analysis

To gain insight into the stability and reactivity of the selected phytochemicals, we performed density functional theory (DFT) calculations with the B3LYP functional and the 6-31G basis set in the Gaussian 09 software package. This approach allowed us to extract key molecular descriptors, including the highest occupied molecular orbital (HOMO) and lowest unoccupied molecular orbital (LUMO) energies, ionization potential, electron affinity, electronegativity, chemical potential, hardness, softness, and electrophilicity index. Visualization of the LUMO and HOMO frontier molecular orbitals was also performed to complement the analysis.^32^, ^33^, ^34^ The electron affinity (A) and ionization potential (I) were calculated with equations (1), (2):

(1) *I* = -*E*_*HOMO*_

(2) *A* = -*E*_*LUMO*_

The chemical potential (*μ*), electronegativity (*χ*), hardness (*η*), softness (*S*), and electrophilicity index (*ω*) were calculated with equations (3), (4), (5), (6), (7), respectively:

(3) $$-\frac{\left(I+A\right)}{2}$$

(4) $$\frac{\left(I+A\right)}{2}$$

(5) $$\frac{\left(I-A\right)}{2}$$

(6) $$\frac{1}{2\eta }$$

(7) $$\frac{\mu^{2}}{2\eta }$$

### Molecular dynamics simulation

We used molecular dynamics (MD) simulations to evaluate the stability of the protein-ligand complexes and to compare the binding behaviors of our identified lead compounds versus established drugs. MD simulations were performed in YASARA Structure software. Before initiation of the simulations, all complexes underwent energy minimization to eliminate unfavorable steric interactions and ensure proper covalent geometry within the molecules. This minimization step helped establish a stable starting point for the simulations. YASARA offers a streamlined automation process through the md_run.mcr macro, which encompasses all simulation stages—preparation, minimization, equilibration, and production—within a single script, thus enhancing computational efficiency. Specific simulation parameters were configured, including a pH of 4.5 to mimic the acidic lysosomal environment of PPT1, a water solvent density of 0.997 g/mL at 310 K, and a physiological ionic solution containing 0.9 % NaCl. This physiological mimicry reflects the operational environment of PPT1 in lysosomes, which is acidic and maintains a temperature similar to that of the human cytoplasm (approximately 310 K).^35^ Furthermore, YASARA facilitated the optimization of the hydrogen bond network during the initial simulation setup, thereby enhancing the stability of the solvated complex. The MD simulation was executed for 500 ns under the AMBER14 force field for parameter calculations with a cutoff of 20 Å. The simulation process with varying temperatures prompted us to remove the "#" sign in the “pressurectrl = ‘Manometer1D, Pressure = 1’” macro section. Trajectory analysis tools provided by the YASARA macro md_analyze.mcr were subsequently used to evaluate various parameters, including root mean square deviation (RMSD), root mean square fluctuation (RMSF), solvent-accessible surface area (SASA), and radius of gyration (Rg). To complement the structural analysis, the binding free energies of the protein-ligand complexes were calculated with the molecular mechanics with Poisson-Boltzmann and surface area solvation (MM-PBSA) method, one of the most reliable end-point approaches for estimating binding free energies in protein-ligand interactions.^36^ The binding free energy was estimated with the following equation:

$$\text{Binding}\;e\text{nergy}=E_{\text{potRecept}}+E_{\text{solvRecept}}+E_{\text{potLigand}}+E_{\text{solvLigand}}-E_{\text{potComplex}}-E_{\text{solvComplex}}$$

## Results

### Validation of PPT1 structure

**Figure 2:**
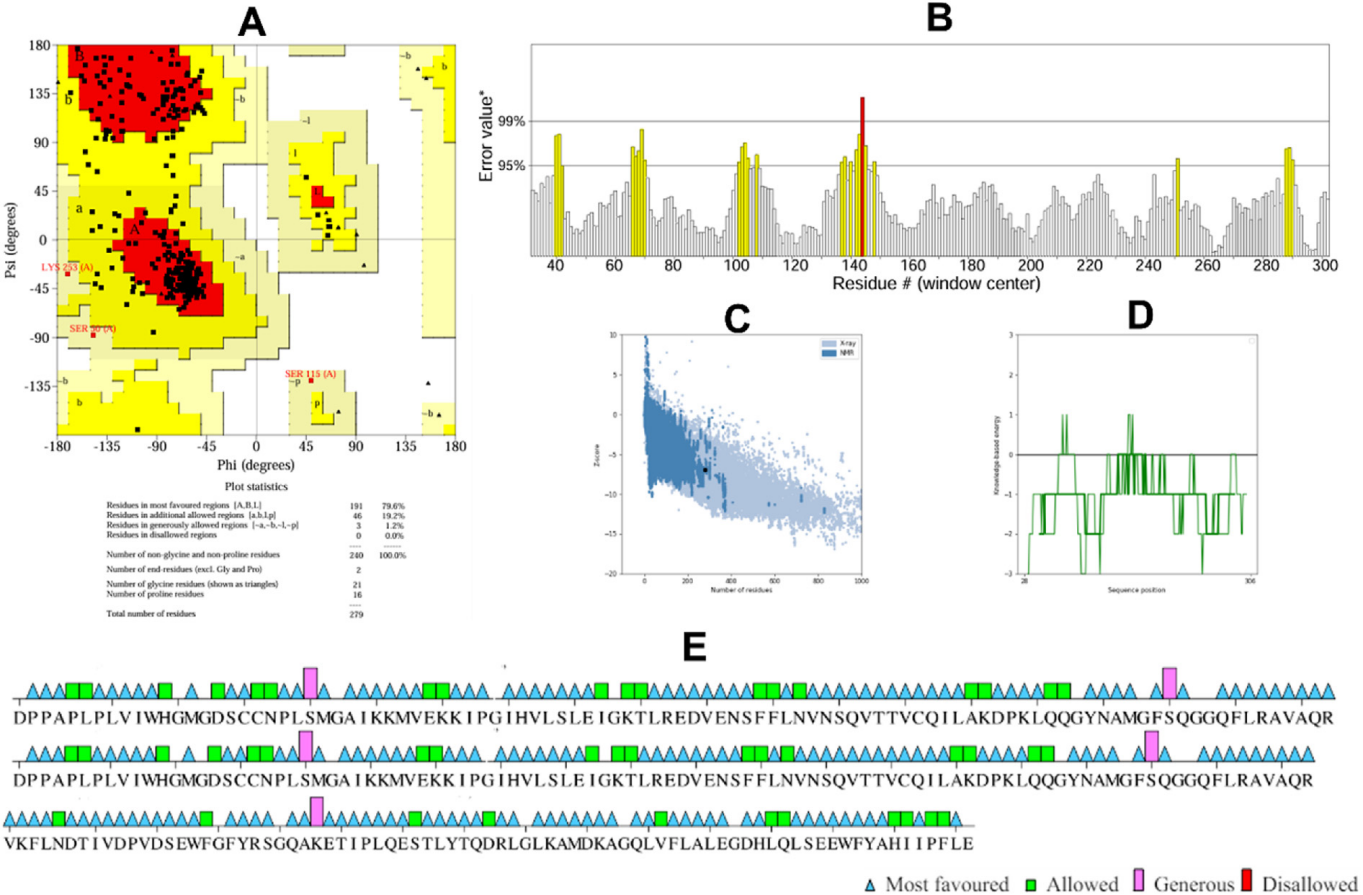
Structural analysis and evaluation of the palmitoyl protein thioesterase 1 (PPT1) enzyme from the enzyme preparation results. The analysis and evaluation were performed with UCLA-DOE LAB–SAVES v6.0 and ProSA-web. A) Ramachandran plot. B) ERRAT. C) Overall model quality. D) Local model quality. E) Sequence and Ramachandran regions.

We first validated the PPT1 receptor structure by assessing the quality of the preparation conducted with YASARA. Ramachandran plot results indicated 79.6 % of residues in the most favored regions, 19.2 % of residues in additional allowed regions, 1.2 % of residues in generously allowed regions, and 0.0 % of residues in disallowed regions (Figure 2A). These results correlated with those from an ERRAT analysis conducted to determine the quality of the PPT1 enzyme (Figure 2B). The value of 90.77 % indicated excellent enzyme quality. Furthermore, the confirmation was obtained through analysis of overall model quality (Figure 2C) and local model quality (Figure 2D) with the ProSA-web server. The PPT1 score of −6.98 and the local model quality graph confirmed the enzyme's excellent overall model quality. Our final analysis revealed that the confirmed sequence was located predominantly in the most favored, allowed, and generous regions, rather than the disallowed region (Figure 2E).

**Figure 3:**
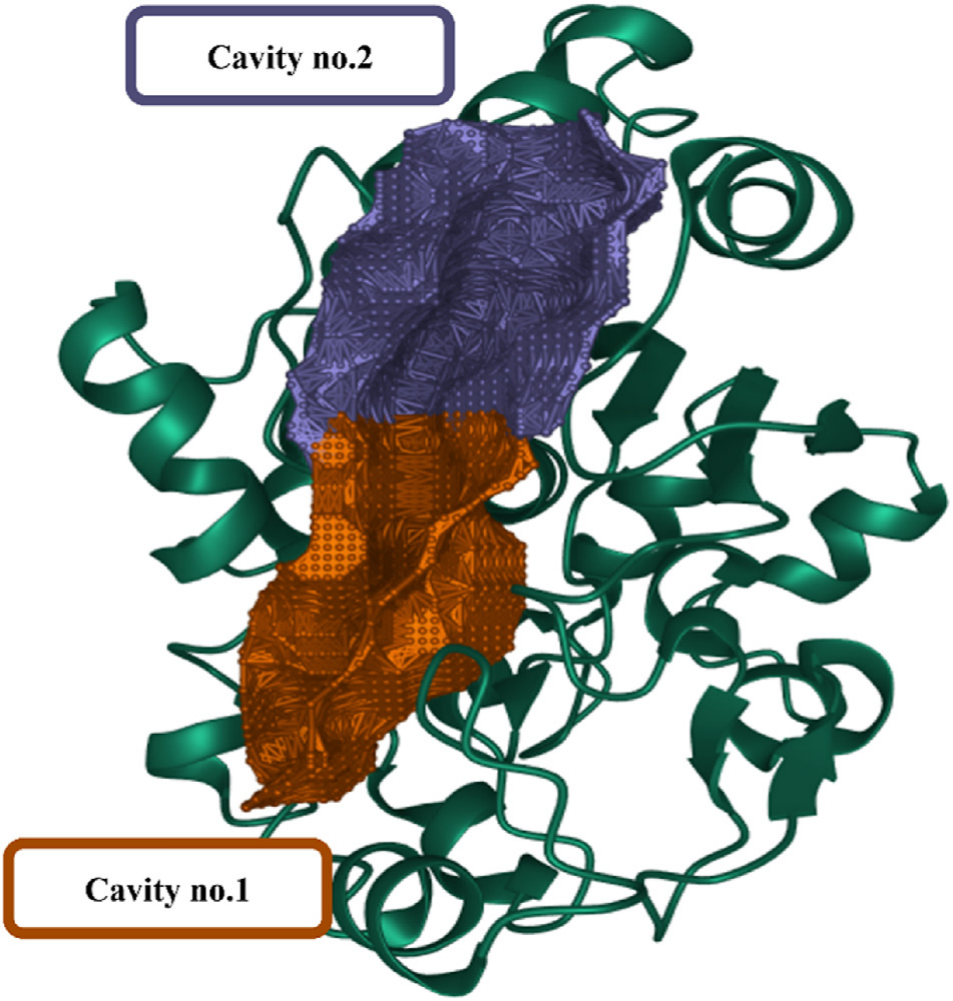
Illustration of two druggable cavity pockets of PPT1.

Pocket site predictions were performed to identify the optimal binding site for ligand inhibition of PPT1. Targeting a suitable pocket within the enzyme structure is crucial for ligand efficacy, by ensuring proper positioning to inhibit protein function. Three cavities were identified with computational tools (Table 1). Cavity 2 emerged as the most promising candidate, because of its high druggability score (1881) indicating favorable ligand binding. Additionally, cavity 2 had the largest volume (803.62 Å³) among the detected cavities while maintaining a comparable surface area (Table 2). Interestingly, most of the investigated ligands interacted with residues in cavity 1, whereas only several ligands interacted with residues in cavity 2 during the simulations (Figure 3). Cavities 1 and 2 had high predicted druggability scores (above 1000) indicating their potential as ligand-binding sites. The specific residues surrounding cavity 1, identified in Figure 3, were considered crucial for further elucidating protein-ligand interactions.

**Table 1:** Druggability prediction of the PPT1 binding pocket with CavityPlus.

Cavity No.	DrugScore	Druggability
1.	1036.00	Strong
2.	1881.00	Strong
3.	−984.00	Weak

**Table 2:** Pocket site predictions.

Cavity No.	Surface area (Å^2^)	Volume (Å^3^)	Residues
1	464.50	634.25	VAL-146, GLY-42, GLY-170, ILE-163, GLN-119, GLN-182, GLY-148, GLU-242, TYR-172, HIS-289, LEU-149, SER-115, ASP-288, HIS-39, GLN-142, ARG-164, VAL-236, PHE-114, ASP-237, LEU-180, ASN-168, GLN-116, PHE-147, ILE-176, LEU-290, SER-83, MET-41, GLY-40, GLY-140, VAL-181, PHE-84, ALA-183, ASP-43, GLU-184, TRP-186, THR-234, GLN-291, TYR-185, GLN-177, ASP-233, ALA-171, LEU-167, ILE-235, PRO-150
2	459.25	803.62	GLY-42, VAL-139, TYR-298, MET-57, HIS-289, PRO-48, SER-115, ASP-288, TRP-38, GLN-142, SER-44, VAL-236, GLU-294, PHE-114, SER-50, GLU-295, GLN-116, GLY-52, LYS-60, LEU-290, ALA-53, SER-293, MET-41, GLY-40, LEU-49, PHE-84, ASP-43, ILE-54, PHE-230, GLN-291, LEU-292, MET-51, TYR-185, LYS-56, ASN-47, ILE-235, HIS-39, PHE-297

**Figure 4:**
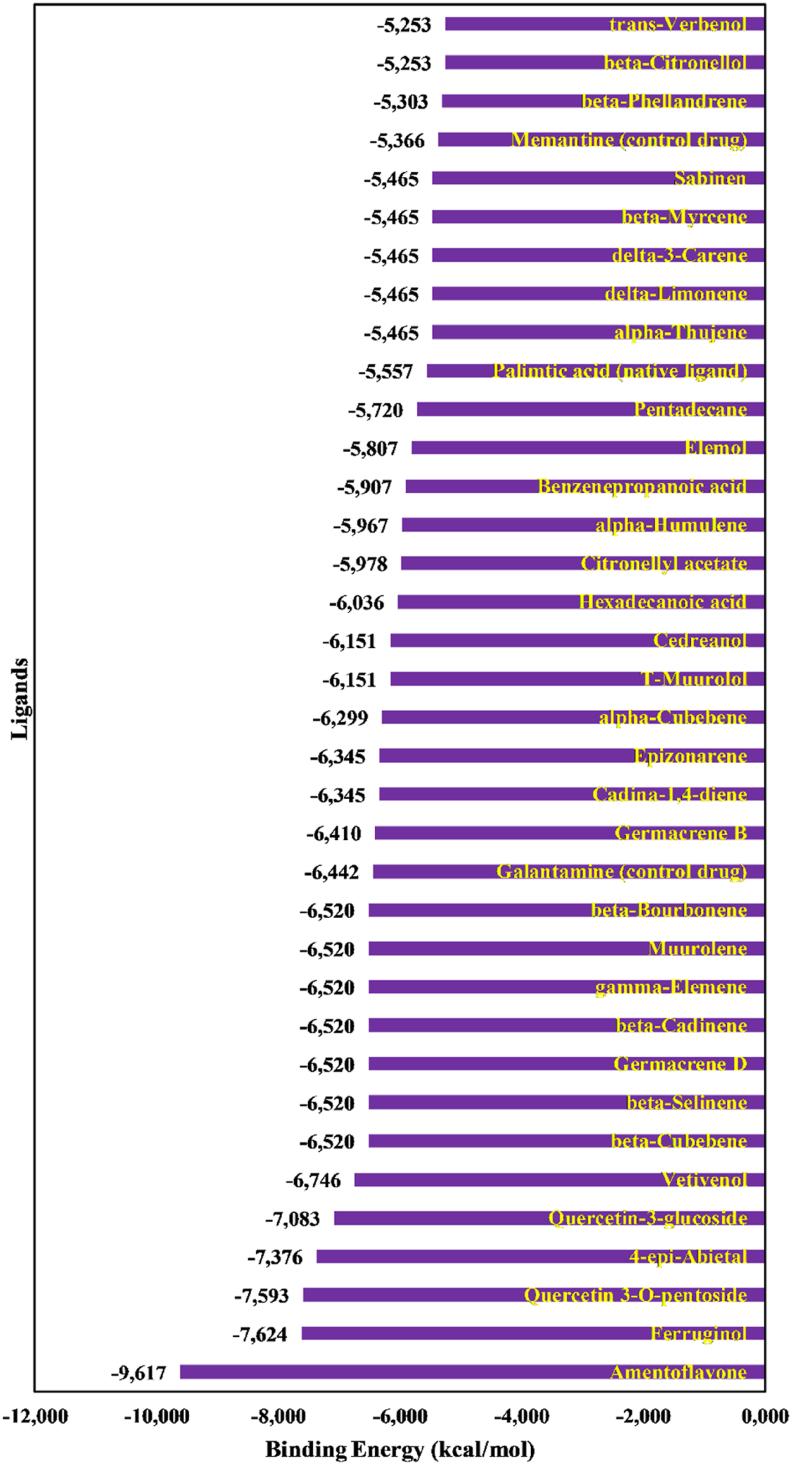
Predicted binding energies of phytocompounds with PPT1.

**Figure 5:**
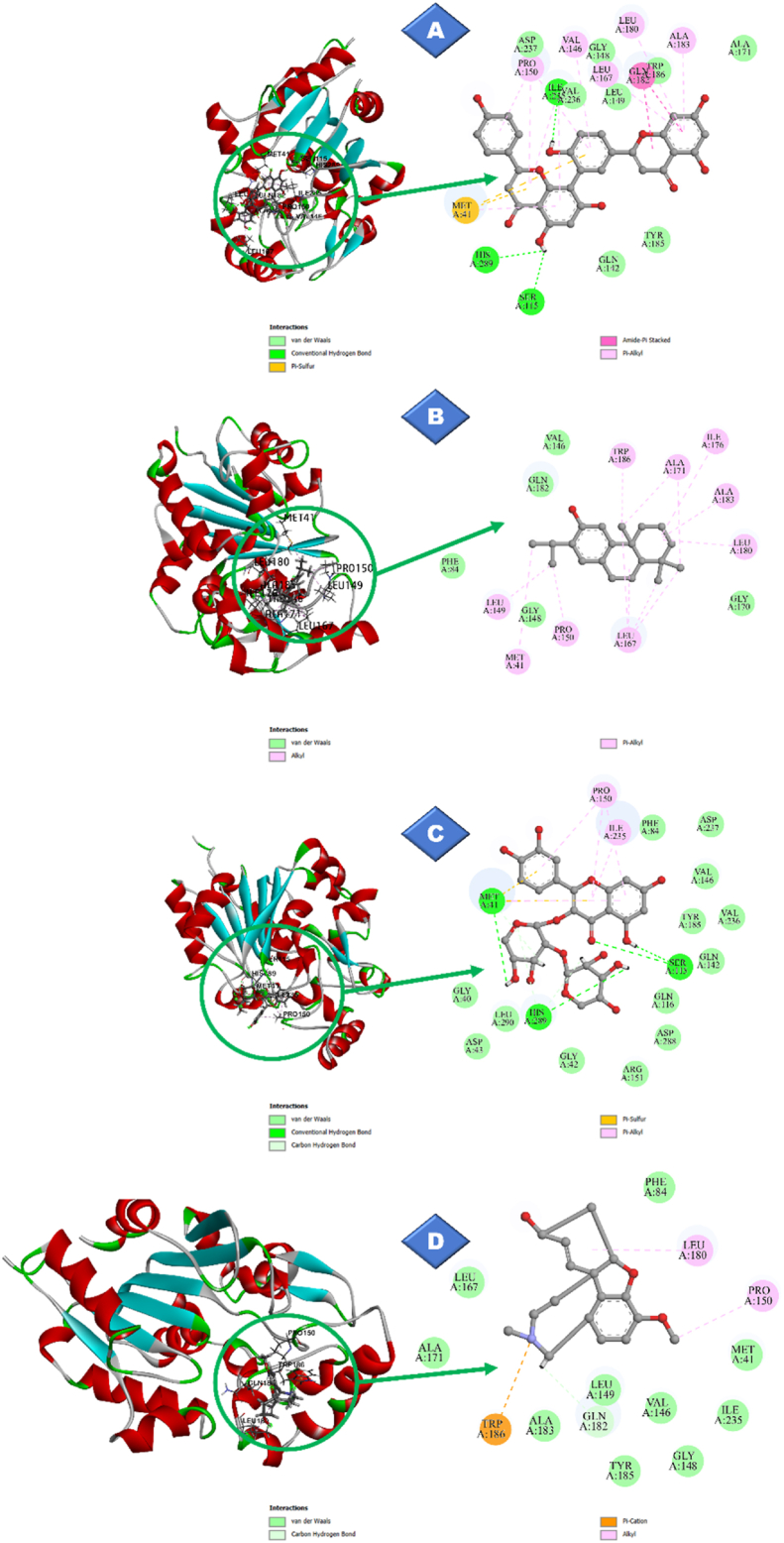
Docking poses and interactions: A) amentoflavone; B) ferruginol; C) quercetin; 3-*O*-pentoside; D) galantamine.

We investigated the potential of 33 phytochemicals to inhibit PPT1 enzymatic activity through *in silico* docking simulations. The reference compounds such as galantamine, memantine, and palmitic acid were included as controls for comparison. The docking results revealed that binding energies ranged from −5.253 kcal/mol (trans-verbenol) to −9.617 kcal/mol (amentoflavone) (Figure 4). The three phytochemicals with the highest binding energies, amentoflavone (−9.617 kcal/mol), ferruginol (−7.624 kcal/mol), and quercetin 3-*O*-pentoside (−7.593 kcal/mol), were selected for further analysis. Analysis of the protein-ligand complexes for these top phytochemicals and the top-ranked control drug, galantamine (according to docking scores), revealed key interaction patterns (Figure 5). The reference compound galantamine formed 14 interactions, including one hydrogen bond, three hydrophobic interactions, and ten van der Waals interactions. Notably, the most promising phytochemical, amentoflavone, exhibited the most interactions (19), consisting of three hydrogen bonds, eight hydrophobic interactions, and eight van der Waals interactions. Ferruginol formed 14 interactions (9 hydrophobic interactions and 5 van der Waals interactions), whereas quercetin 3-*O*-pentoside formed 18 interactions (3 hydrogen bonds, 2 hydrophobic interactions, and 13 van der Waals interactions). Further investigation of specific interactions revealed that amentoflavone formed hydrogen bonds with Ser^115^, His^289^, and Ile^235^ residues. Similarly, quercetin 3-*O*-pentoside formed hydrogen bonds with the Ser^115^ and His^289^ residues. In contrast, galantamine formed only a hydrogen bond with Gln^182^. Interestingly, the residues Met^41^, Val^146^, and Pro^150^ emerged as key binding sites, because all ligands interacted with these residues.

Selected phytochemicals were further evaluated for toxicity according to the Pfizer rule and Protox 3.0 web server. Protox 3.0 predicted the compounds' LD_50_, toxicity class, hepatotoxicity, neurotoxicity, mutagenicity, cytotoxicity, and carcinogenicity. Amentoflavone and quercetin 3-*O*-pentoside showed LD_50_ values of 3919 and 5000 mg/kg, respectively, and were classified in toxicity class 5. Ferruginol had an LD_50_ of 2000 mg/kg and was classified in toxicity class 4. No compounds showed any activity in the hepatotoxicity, neurotoxicity, mutagenicity, cytotoxicity, and carcinogenicity assays. In contrast, the control drug had a low toxicity class (3) and low LD_50_ values of 85 mg/kg, and showed neurotoxic and cytotoxic activity. The Protox 3.0 results indicated that all ligands were safe except galantamine (Table 3). However, ferruginol had a lower LD_50_ value and did not comply with the Pfizer rule, and therefore might be more toxic than the other two compounds. Admetlab 2.0 was used to assess further the ligands’ safety according to the Pfizer rule. The parameters used were TPSA and lipophilicity (logP). Ligands with low TPSA (<75) and high logP (>3) are considered potentially toxic. The Admetlab 2.0 results indicated that ferruginol and galantamine violated the Pfizer rule, with TPSA values of 20.2 and 41.93, respectively (Table 3). Therefore, ferruginol was not selected for further evaluation with the PASS server.

**Table 3:** Toxicity and Pfizer rule properties.

Ligand	Protox 3.0							Pfizer rule	
	Predicted LD_50_ (mg/kg)	Predicted toxicity class	Hepatotoxicity	Neurotoxicity	Mutagenicity	Cytotoxicity	Carcinogenicity	TPSA	logP
Amentoflavone	3919	5	Inactive	Inactive	Inactive	Inactive	Inactive	181.8	3.0
Ferruginol	2000	4	Inactive	Inactive	Inactive	Inactive	Inactive	20.2	6.1
Quercetin 3-*O*-pentoside	5000	5	Inactive	Inactive	Inactive	Inactive	Inactive	249.2	−0.5
Galantamine (control drug)	85	3	Inactive	Active	Inactive	Active	Inactive	41.930	1.389

Amentoflavone and quercetin 3-*O*-pentoside were further evaluated with the PASS server to predict their Pa in dementia treatment. This *in silico* approach targeted dementia treatment according to the established role of PPT1 in neuronal health and its links with neurodegenerative diseases. Both ligands exhibited a higher Pa than Pi. Interestingly, quercetin 3-*O*-pentoside displayed a greater Pa value (0.423) than amentoflavone (0.287), thus suggesting its potentially higher efficacy for dementia treatment. Amentoflavone and quercetin 3-*O*-pentoside have different chemical structures. In contrast, galantamine showed a better Pa value than quercetin 3-*O*-pentoside (0.458), possibly because quercetin 3-*O*-pentoside has functional groups enabling more favorable interactions with PPT1, the target protein involved in neuronal health.

**Figure 6:**
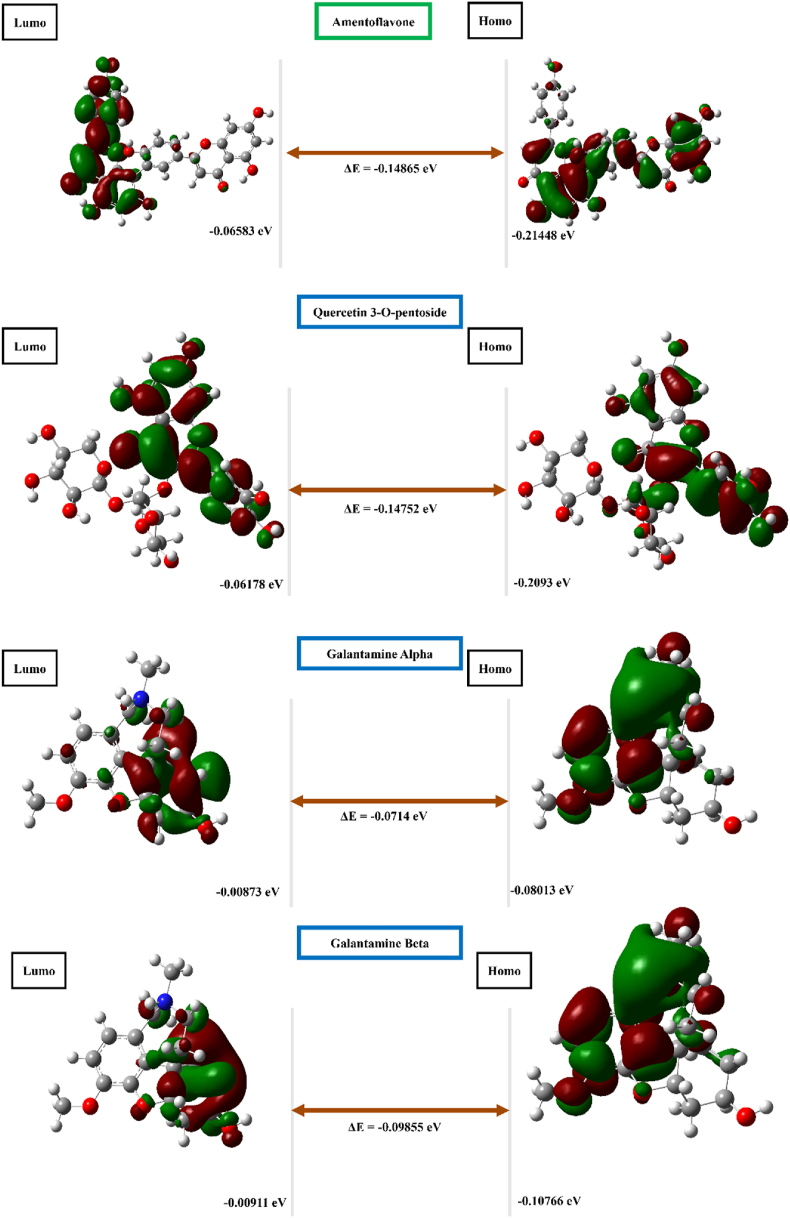
Visualization of the frontier molecular orbitals of selected compounds through calculations with B3 LYP/6-31G (d,2p).

To identify promising phytochemicals, DFT is required; this well-established quantum mechanical approach has been lauded for its precision in revealing both structural and electronic properties of compounds. By leveraging the B3LYP/6-31G∗ level of theory implemented in Gaussian 09W software, we carefully optimized the molecular structures of the investigated phytochemicals with the B3LYP 6-31G (D, 2P) basis set. This powerful method facilitated in-depth understanding of the electronic characteristics governing these molecules. To comprehensively map the electron density distribution across the phytochemicals, we computed the HOMO and LUMO (visualized in Figure 6, in which green and red hues indicate positive and negative phases of the electron density, respectively). Furthermore, Table 5 presents a comprehensive analysis encompassing the calculation of ionization potential, electron affinity, chemical potential, electronegativity, hardness, softness, and electrophilicity index. We observed strikingly similar values for our two most promising phytochemical candidates (Table 5). The candidates and galantamine also exhibited favorable characteristics of low hardness coupled with high softness. Remarkably, amentoflavone surpassed quercetin 3-*O*-pentoside and galantamine in all evaluated parameters, thus supporting its potential as a superior phytochemical.

**Table 4:** Prediction of the bioactivity of each ligand with PASS online.

Ligand	Dementia treatment	
	Pa	Pi
Galantamine (control drug)	0.458	0.026
Amentoflavone	0.287	0.16
Quercetin 3-*O*-pentoside	0.423	0.04

**Table 5:** Density functional theory calculations.

Ligand		HOMO (eV)	LUMO (eV)	Energy gap (eV)	Ionization potential (I) (eV)	Electron affinity (A) (eV)	Chemical potential (μ) (eV)	Electronegativity (χ) (eV)	Hardness (η) (eV)	Softness (S) (eV)	Electrophilicity index (ω) (eV)
Amentoflavone		−0.21448	−0.06583	−0.14865	0.21448	0.06583	−0.14016	0.14016	0.07433	6.72721	0.13215
Quercetin 3-*O*-pentoside		−0.20930	−0.06178	−0.14752	0.20930	0.06178	−0.13554	0.13554	0.07376	6.77874	0.12453
Galantamine (control drug)	Alpha	−0.08013	−0.00873	−0.0714	0.08013	0.00873	−0.04443	0.04443	0.0357	14.00560224	0.027647408
	Beta	−0.10766	−0.00911	−0.09855	0.10766	0.00911	−0.058385	0.058385	0.049275	10.14713343	0.034589632

MD simulations lasting 500 ns, a valuable tool in drug discovery, allowed us to investigate the time-dependent behavior of biomolecular systems of amentoflavone and galantamine in PPT1 and assess binding stability. We monitored various parameters throughout the MD simulations to acquire detailed information about protein dynamics and ligand–protein interactions. These parameters included backbone RMSD (RMSD_backbone_), RMSF, Rg, SASA, and calculation of molecular mechanics Poisson–Boltzmann surface area (MM-PBSA). The results indicated that amentoflavone was superior to galantamine in all investigated aspects.

## Discussion

Dementia, a condition characterized by progressive cognitive decline, markedly affects individuals, families, and healthcare systems worldwide. Dementia medications have limitations, and their use should be approached with caution. These limitations include adverse effects, interactions with other drugs, and requirements for frequent regimen adjustments. Additionally, the effectiveness of these medications diminishes over time, and some patients dislike them. Physicians must consider underlying conditions, potential adverse effects of combination treatments, and alternative treatments before prescribing. Finally, medication may be discontinued if the benefits are minimal or outweighed by adverse effects.^37^ Therefore, effective new drugs must be developed to combat this disease. To avoid the time-consuming, labor-intensive trial-and-error approach, we screened, designed, and predicted the biological activity of potent *J. phoenicea* derivatives, according to their chemical structures.^38^ Furthermore, we validated the initial analysis of the quality of the PPT1 enzyme preparation procedure, on the basis of Ramachandran plot analysis, ERRAT analysis, overall model quality, local model quality, and sequence. These analyses confirmed that our preparation did not adversely affect the quality of the PPT1 enzyme.

PPT1 is an excellent target for anti-dementia therapy, because of its critical role in maintaining protein homeostasis and its association with neurodegenerative diseases.^39^ Identifying potential binding sites with favorable characteristics is crucial in drug discovery and optimization before the docking process.^40^ In this study, we conducted a comprehensive computational investigation of the inhibition of PPT1, through a 500 ns MD simulation. Although the neuroprotective properties of *J. phoenicea* L. have been established, its molecular mechanism of action, particularly its interaction with PPT1, has not been fully elucidated. Our results provide a novel computational framework for targeting PPT1 and offer insights into the potential of *J. phoenicea* as a primary source for drug discovery. Software was used to analyze the target protein and identify suitable cavities. Cavity 1 was selected for further investigation, on the basis of its high druggability score (above 1000) suggesting its potential to accommodate small molecule ligands. The cavity score provides valuable information regarding the potential druggability of the cavity, which can be used to guide the design and optimization of small molecules that bind the cavity and modulate the target protein's function.^41^ In this scenario, cavity 1 had a lower druggability score and smaller volume, but all ligands bound the residues in this cavity. Therefore, cavity 1 might still be a viable target for drug development, because of its demonstrably high propensity for ligand binding and potential modulation of PPT1 function. Additionally, the ligand occupancy of cavity 1 underscores its promise as a target for further drug design efforts (Figure 3). In contrast, cavity 2 had a higher druggability score and larger volume, but ligands did not bind some residues of this cavity. This finding might have been because the specific residues in cavity 2 were not suitable for ligand binding, or suitable ligands for these residues were lacking. However, cavity 2's higher druggability score and larger volume make it a potentially valuable target for drug discovery. In conclusion, on the basis of the observed ligand–protein interactions, cavity 1 might be a suitable starting point for researchers exploring PPT1-targeting ligands.

We conducted docking simulations to evaluate the binding interactions between all 33 phytochemicals and PPT1. The binding energies ranged from −5.253 kcal/mol to −9.617 kcal/mol, and 13 ligands displayed more favorable binding energies than the reference compound galantamine (−6.442 kcal/mol). Notably, amentoflavone emerged as the ligand with the most favorable binding energy. These results suggested the promising potential of these phytochemicals for PPT1 inhibition. Amentoflavone notably demonstrated superior binding affinity to the control drug, as evidenced by its more favorable docking score (Figure 4). Therefore, amentoflavone might serve as a promising candidate for further investigation. In addition, quercetin 3-*O*-pentoside and ferruginol ranked second and third, respectively, and warrant further investigation, given their promising docking scores.

This study was aimed at predicting the potential human toxicity of two selected compounds. However, our analysis relied solely on *in silico* predictions based on Protox 3.0 and the Pfizer rule implemented within ADMETLab 2.0. Protox 3.0 offers insights into various toxicity-related parameters (Table 3). This server provides a valuable tool for streamlining the drug design process by facilitating the prediction of compound toxicity. Notably, computational toxicity prediction provides a more efficient and ethical alternative to traditional animal testing.^29^ The predictions indicated that three investigated compounds (amentoflavone, ferruginol, and quercetin 3-*O*-pentoside) exhibited non-toxic profiles, whereas galantamine did not (Table 3). Furthermore, we incorporated the Pfizer rule to complement our toxicity assessment. This rule serves as a rapid filtering tool for identifying phytocompounds with favorable absorption, distribution, metabolism, and excretion (ADME) properties. The rule posits that compounds with lower logP values (indicating greater polarity) and higher TPSA values (indicating greater hydrophilicity) are more likely to demonstrate optimal ADME characteristics. All three compounds resided within the "desirable" region defined by the Pfizer rule, thus suggesting their potentially favorable oral bioavailability.^42^ However, on the basis of the TPSA values, ferruginol and galantamine appeared to be outliers with respect to the other two compounds, given their lower TPSA values (Table 3). This finding prompts potential toxicity concerns and warrants exclusion of galantamine from further analysis.

We used the PASS server to predict the Pa of amentoflavone and quercetin 3-*O*-pentoside in dementia treatment. The PASS server's designation of PPT1 as a dementia treatment is likely to be based on the understanding of the role of PPT1 in neuronal health and its association with neurodegenerative disorders. Targeting PPT1 might enable modulation of the protein palmitoylation/depalmitoylation balance, which has been implicated in AD and other neurodegenerative conditions, thus leading to the development of therapeutic strategies for treating neurodegenerative diseases.^20^ The results indicated that amentoflavone and quercetin 3-*O*-pentoside are Pa > Pi (Table 4). High differences between values indicate favorable probability of biological activity.^43^ However, because quercetin 3-*O*-pentoside had a higher Pa value (0.423) than amentoflavone (0.287), the presence of a pentoside sugar group in quercetin 3-*O*-pentoside might contribute to its favorable interactions with PPT1. The sugar group might provide additional hydrogen bonding sites or other non-covalent interactions that enhance the binding affinity of quercetin 3-*O*-pentoside to PPT1. Additionally, the presence of a 3-*O*-pentoside group might also enable more favorable interactions with other proteins or cellular components involved in neuronal health, thus contributing to this compound's potential efficacy in treating dementia.

DFT calculations were used to elucidate protein-ligand interactions and assess the inhibitory potential of the investigated phytocompounds, as well as their reactivity and stability. The energy gap, defined as the difference in energy between the HOMO and LUMO levels, provides insights into compound stability. Amentoflavone exhibited a smaller energy gap (−0.14865 eV) than quercetin 3-*O*-pentoside (−0.14752 eV) and galantamine (−0.714 eV and −0.09855 eV) (Table 5). A narrower energy gap generally translates to stronger bonding and facilitates electronic transitions.^32^^,^^44^ This finding is consistent with our docking results suggesting that amentoflavone might possess superior binding affinity. Galantamine has two locations of HOMO and LUMO orbitals, because of the presence of multiple molecular orbitals with different energies. This phenomenon might be attributable to the complex structure with multiple conjugated systems or multiple types of atoms and functional groups in the molecule.^45^ All investigated ligands displayed greater softness than hardness (Table 5). Our calculations suggested that the compounds might exhibit electrophilic behavior but also possess relatively low reactivity, thereby leading to thermodynamic stability. This possibility was further supported by the HOMO/LUMO gap and the better chemical potential values. The softness values of amentoflavone (6.72721 eV) and quercetin 3-*O*-pentoside (6.77874 eV) indicated their inherently low reactivity, whereas galantamine's higher value indicated its reactivity (14.0056 eV and 10.1471 eV). Additionally, the electrophilicity index (ω) suggested that these compounds are borderline electrophiles, with values near the limit of ω < 0.8 eV.^45^ The negative chemical potential (μ) values observed for both compounds suggested good stability and potential formation of stable complexes with the target receptor (Table 5). Collectively, these findings suggested that amentoflavone and quercetin 3-*O*-pentoside have promise as candidates for anti-dementia therapeutics. On the basis of the combined results from docking and DFT studies, we selected amentoflavone for further exploration through MD simulations.

**Figure 7:**
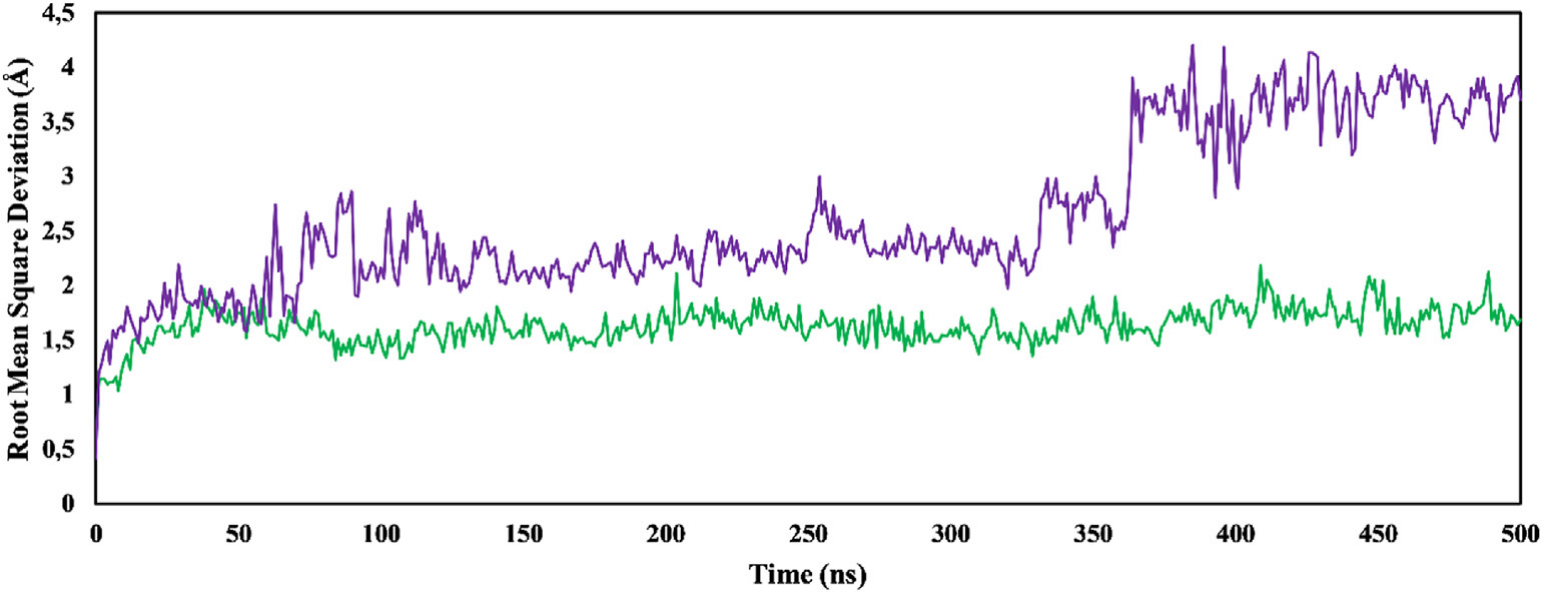
Changes in root mean square deviation values of ligand complexes. Amentoflavone-PPT1 (green); galantamine-PPT1 (purple).

**Figure 8:**
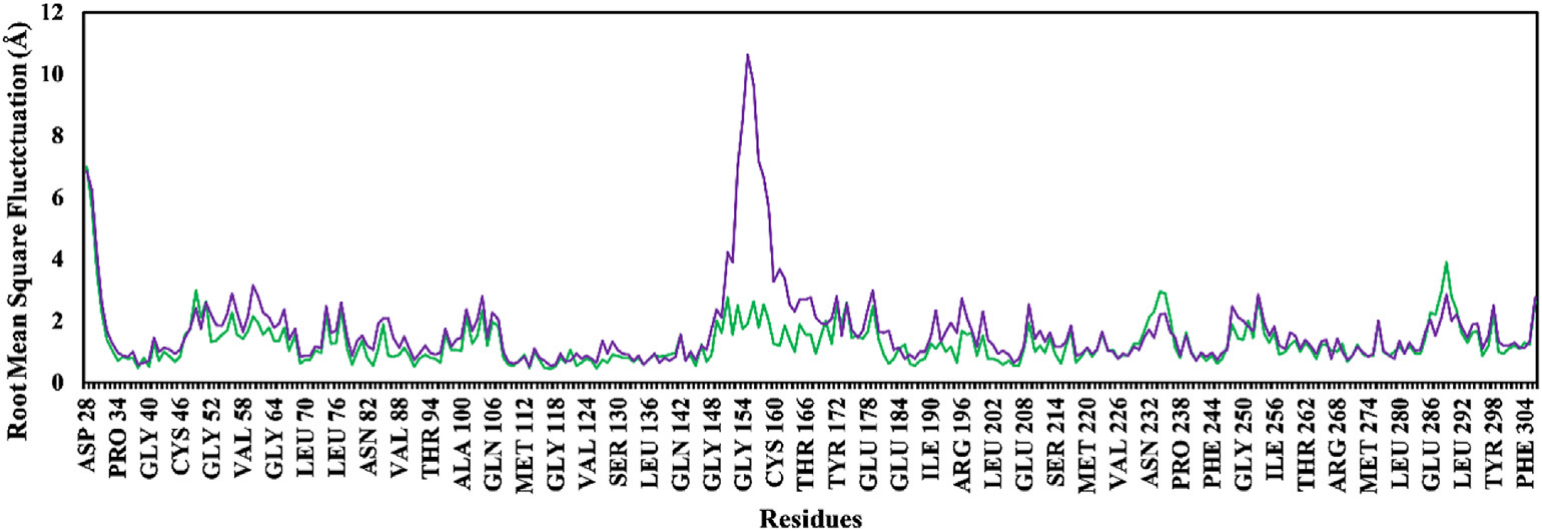
Changes in root mean square fluctuation values of ligand complexes. Amentoflavone-PPT1 (green); galantamine-PPT1 (purple).

An MD simulation for 500 ns was conducted to explore the behavior at the binding side and determine the stability of the complex. The results indicated that amentoflavone was superior to galantamine, the control dementia drug, and excelled according to all studied parameters. This method provides a more statistically robust picture of protein-ligand binding than docking calculations in terms of the cellular aqueous environment, electroneutrality, temperature, pressure, and volume. The first parameter, RMSD_backbone_, provides information on stability and dynamic behavior over a 500 ns time scale. After an initial period of minor fluctuations attributed to kinetic shock, all systems reached steady state. Kinetic shock is frequently observed in MD simulations. Amentoflavone-PPT1 exhibited greater stability than galantamine-PPT1 (Figure 7). The average RMSD for amentoflavone-PPT1 was 1.63 Å, a value substantially lower than the 2.62 Å observed for galantamine-PPT1. Complexes with an average RMSD exceeding 2.5 Å are generally considered unstable.^46^ Notably, amentoflavone-PPT1 achieved equilibrium within 48 ns and maintained stability throughout the simulation. In contrast, galantamine-PPT1 displayed periods of instability between 98 and 250 ns and again between 260 and 330 ns, thus further supporting its lower overall stability. In agreement with the RMSD_backbone_ data, the RMSF plot (Figure 8) demonstrated lower average fluctuations and flexibility for individual residues in amentoflavone-PPT1 than galantamine-PPT1. The RMSF value quantified the average fluctuation of each residue within the protein structure.

**Figure 9:**
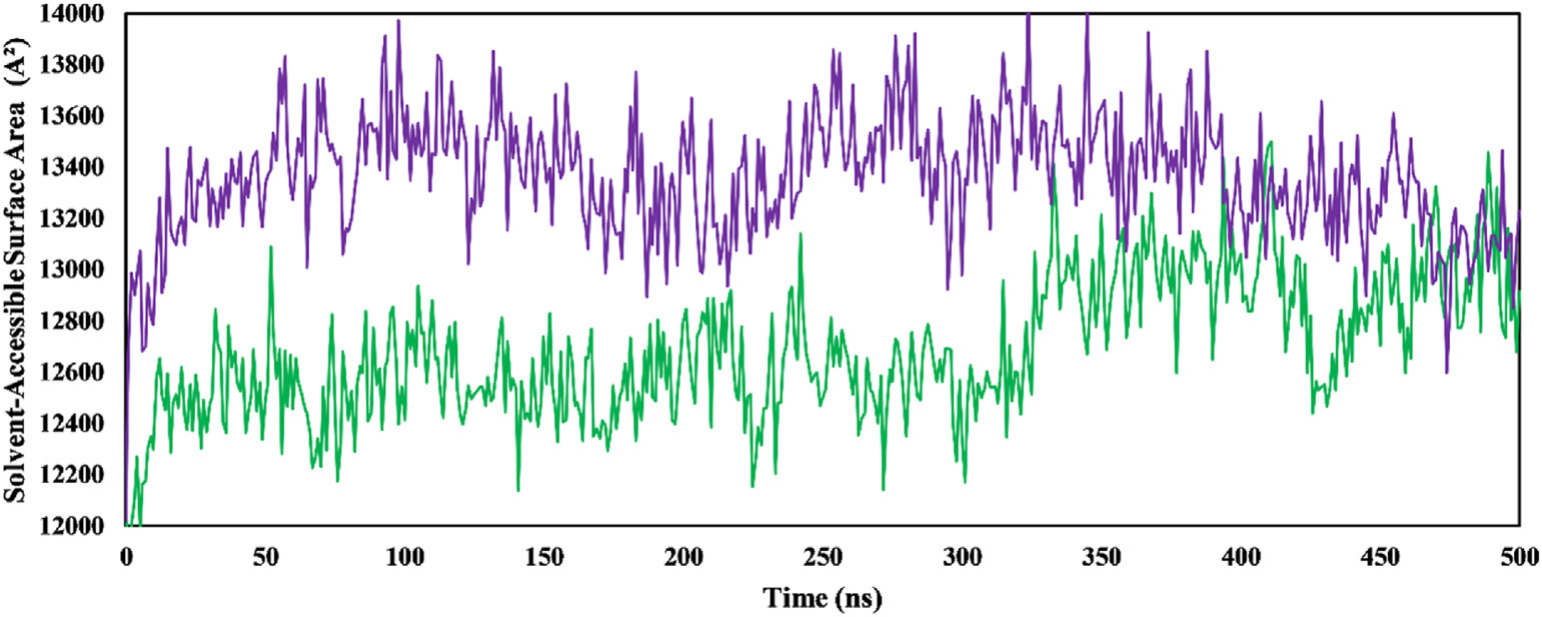
Changes in solvent-accessible surface area values of ligand complexes. Amentoflavone-PPT1 (green); galantamine-PPT1 (purple).

SASA reflects changes in the solvent interaction profile of a protein after ligand binding. We used SASA to evaluate the complexes’ interaction with the surrounding solvent environment over the 500 ns simulation trajectory. The results depicted in the graph indicated that amentoflavone-PPT1 displayed a lower average SASA value (12688.18 Å^2^) than galantamine-PPT1 (13365.85 Å^2^). Interestingly, neither complex exhibited significant expansion or contraction of the SASA throughout the simulation (0–500 ns), thereby suggesting minimal conformational changes after ligand binding (Figure 9).

**Figure 10:**
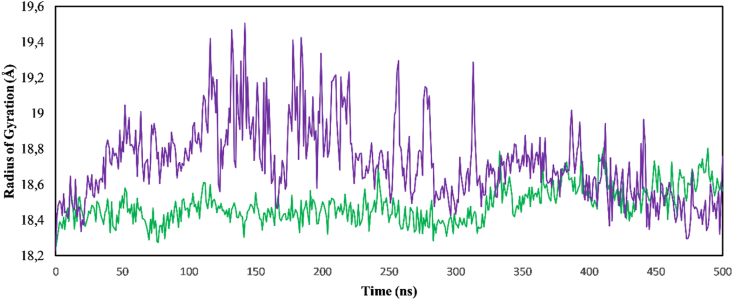
Changes in radius of gyration values of ligand complexes. Amentoflavone-PPT1 (green); galantamine-PPT1 (purple).

The Rg provides insights into the equilibrium conformation, compactness, and folding behavior of proteins and protein-ligand complexes. Analysis of the graph in Figure 10 indicated that amentoflavone-PPT1 maintained a stable Rg value throughout the entire 500 ns simulation, thus indicating a well-defined and compact structure. In contrast, galantamine-PPT1 exhibited Rg fluctuations suggesting initial difficulties in achieving stability. Whereas galantamine-PPT1 did show a period of stability between 335 ns and 360 ns, this stability was not maintained throughout the simulation. Notably, amentoflavone-PPT1 displayed a lower average Rg value (18.48 Å) than galantamine-PPT1 (18.72 Å), thus indicating a more compact structure. Interestingly, during the final 60 ns of the simulation (440–500 ns), galantamine-PPT1 exhibited a lower Rg value than amentoflavone-PPT1. However, the transient nature of this observation and the overall higher Rg for galantamine-PPT1 suggested that amentoflavone-PPT1 is likely to have superior stability.^47^

**Figure 11:**
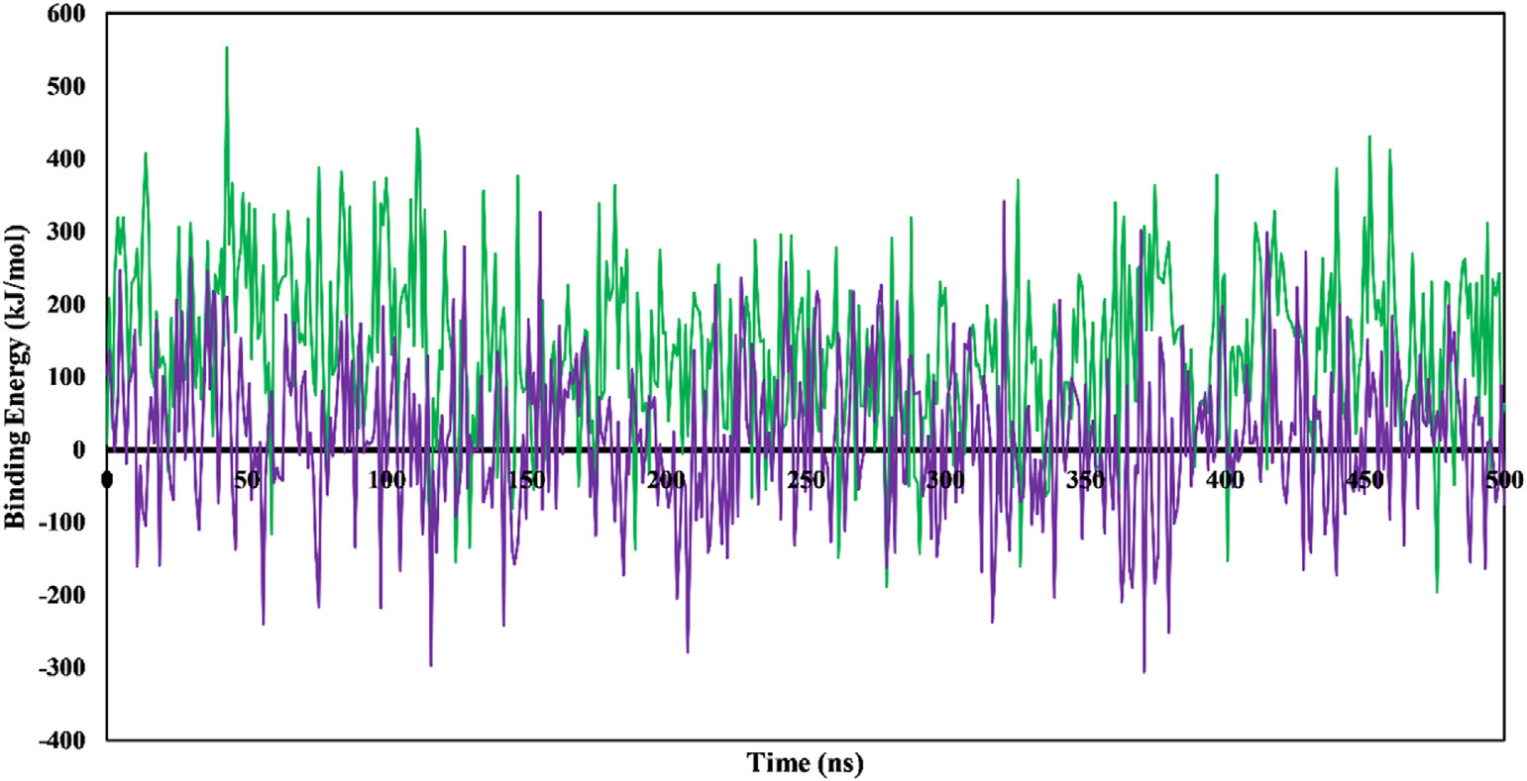
Changes in binding energy values of ligand complexes. Amentoflavone-PPT1 (green); galantamine-PPT1 (purple).

Binding free energy analysis is crucial for estimating the energetics associated with ligand binding PPT1 during MD simulations. Accurate calculation of binding free energies is essential for understanding protein-ligand interactions. In YASARA Structure software, a more positive binding energy value indicates stronger affinity between the ligand and the target protein. MM-PBSA calculations decompose the binding energy into several components, such as van der Waals energy, electrostatic energy, polar solvation energy, nonpolar solvation energy, and desolvation energy. These components aid in understanding the contributions of various factors to the overall binding energy.^48^ We observed that amentoflavone-PPT1 exhibited a superior binding profile to galantamine-PPT1. Amentoflavone formed more favorable interactions with PPT1 (552) than galantamine (296) (Figure 11). Furthermore, amentoflavone-PPT1 displayed a more favorable average binding energy (−139.87 kJ/mol) than the reference compound galantamine-PPT1 (−19.275 kJ/mol). These observations suggested that amentoflavone is likely to have stronger binding affinity than galantamine to PPT1.

In conclusion, our findings suggest that amentoflavone might be a promising candidate for further development as a PPT1 inhibitor. This naturally occurring biflavonoid with a well-established presence in traditional Chinese medicine has a documented history of use for various health conditions. Notably, it exhibits strong anti-cancer, neuroprotective, antiviral, and anti-inflammatory effects. Additionally, it is effective in the treatment of ulcers, psoriasis, and arthritis, thus highlighting its potential therapeutic applications.^49^ To further validate these *in silico* findings, *in vitro* and *in vivo* studies are warranted.

## Conclusions

This study used *in silico* methods to comprehensively evaluate the potential of *J. phoenicea* phytochemicals as novel PPT1 inhibitors for dementia treatment. Our findings highlight amentoflavone, a naturally occurring biflavonoid, as a promising lead compound for further development. Amentoflavone outperformed the reference drug galantamine in docking simulations, exhibiting superior binding energy (−9.6 kcal/mol) and forming more interactions (19) with the PPT1 binding pocket. *In silico* toxicity analysis predicted a favorable safety profile for amentoflavone, in compliance with established criteria such as the Pfizer rule and predictions from the Protox 3.0 server. PASS server analysis suggested a higher Pa for amentoflavone than quercetin 3-*O*-pentoside in dementia treatment. DFT calculations revealed amentoflavone's slightly more favorable electronic properties than those of quercetin 3-*O*-pentoside, thus potentially indicating enhanced stability and reactivity. MD simulations further confirmed amentoflavone's superior binding stability to galantamine. Amentoflavone displayed consistently lower RMSD, RMSF, and Rg values than galantamine—findings indicating a more stable and compact complex throughout the simulation. Notably, amentoflavone-PPT1 exhibited a significantly more favorable binding free energy (−139.87 kJ/mol) than galantamine-PPT1 (−19.275 kJ/mol), thereby suggesting a stronger binding affinity. Our findings demonstrated the potential of amentoflavone as a novel PPT1 inhibitor for dementia treatment. Amentoflavone's favorable binding profile, predicted safety, and superior *in silico* stability with respect to those of the reference drug highlight its promise as a lead compound for further investigation. This study contributes valuable insights into the development of natural product-based therapeutics for neurodegenerative diseases by identifying amentoflavone as a promising candidate for PPT1 inhibition. Further *in vitro* and *in vivo* studies are warranted to validate these findings, and explore amentoflavone's potential for clinical application in dementia treatment.

## Source of funding

This research did not receive any specific grant from funding agencies in the public, commercial, or not-for-profit sectors.

## Ethical approval

Not applicable; there are no ethical issues associated with this work.

## Author contribution

Riyan Alifbi Putera Irsal: Conceptualization; data curation; validation; investigation; project administration; methodology; resources; formal analysis; writing – original draft; writing – review & editing. Gusnia Meilin Gholam: Conceptualization; data curation; validation; investigation; methodology; resources; formal analysis; writing – original draft; writing – review & editing. Maheswari Alfira Dwicesaria: Data curation; resources; validation; investigation; writing – eview & editing. Tiyara Fany Mansyah: Validation; review & editing. Fernanda Chairunisa: Supervision; review & editing. The final draft of the manuscript's content has been reviewed and approved by all authors, who are also accountable for its content. All authors have critically reviewed and approved the final draft and are responsible for the content of the manuscript.

## Conflict of interest

The authors have no conflicts of interest to declare.
